# Estimating the effects of Basic Income schemes on mental and physical health among adults aged 18 and above in the UK: A microsimulation study

**DOI:** 10.1371/journal.pmen.0000206

**Published:** 2024-12-18

**Authors:** Howard Robert Reed, Elliott Aidan Johnson, Graham Stark, Daniel Nettle, Kate E. Pickett, Matthew Thomas Johnson

**Affiliations:** 1 Department of Social Work, Education and Community Wellbeing, Northumbria University, Newcastle upon Tyne, United Kingdom; 2 Landman Economics, Colchester, United Kingdom; 3 Virtual Worlds, Milton Keynes, United Kingdom; 4 Institut Jean Nicod, Ecole Normale Supérieure, Paris, France; 5 University of York, York, United Kingdom; University of North Carolina at Chapel Hill, UNITED STATES OF AMERICA

## Abstract

Basic Income is a largely unconditional, regular payment to all permanent residents to support basic needs. It has been proposed as an upstream health intervention by increasing income size and security. Modelling has quantified prospective effects on UK young people’s mental health. This paper extends this analysis to mental and physical health among adults aged 18+ using data from the 2021/22 Family Resources Survey and 12 waves (2009/11-2020/22) of Understanding Society to model the effects of three prospective schemes: 1) (£ per week) £50 per under-18, £75 per 18–64, £205 per 65+; 2) £75, £185, £205; 3) £100, £295, £295. We estimated effects on cases of depressive disorders (SF-12 MCS ≤45.6) and physical health problems (SF-12 PCS ≤50), quality-adjusted life years (QALYs) and willingness-to-pay value gained, as well as direct NHS, personal social services and patients’ associated costs savings regarding depressive disorders. Between 124,000 (95% CI: 86,000–150,000) and 1.005m (95% CI: 845,000–1.402m) cases of depressive disorders and 118,000 (70,000–156,000) to 1.042m (881,000–1.612m) cases of physical health problems could be prevented or postponed each year depending on the scheme. 129,000 (86,000–172,000) to 655,000 (440,000–870,000) QALYs could be gained, valued at £3.87bn (£2.58bn–£5.16bn) to £19.65bn (£13.21bn–£26.10bn). Estimated 2023 NHS and personal social services cost savings are between £126m (£88m–£154m) and £1.026bn (£872m–£1.432bn) assuming 50% of depressive disorders cases are diagnosed and treated at baseline. Estimating savings based on physical health problems is more difficult, but may reflect far greater related NHS and social care spend. Although non-income change impacts are not microsimulated, these findings indicate that Basic Income could provide substantial population health benefits, social return on investment and health and social care system savings. This gives policymakers and researchers an evidence base on which to base trial and policy design. Basic Income; Social determinants; Prevention; Upstream interventions; Microsimulation modelling.

## Introduction

Observational and experimental associations between income disparities and health have been established in studies and reviews examining, for example: self-rated health; [1–4] mortality; [1, 2] biomarkers; [4] child health and wellbeing outcomes; [5] mental health among children and young people; [6–8] and adult mental health [9–11]. Supporting Pickett & Wilkinson’s causal review, [6] Adeline and Delattre’s [12] analysis endorsed both the Absolute Income Hypothesis (a positive and concave effect of income on health) and the Income Inequality Hypothesis (that income inequalities affect the health and wellbeing of nearly all members of a society). A recent review [13] has questioned the Income Inequality Hypothesis, but in controlling for individual level income in a multilevel framework to examine the causal effect of income inequality it may over-control for factors on the causal pathway. The balance of evidence supports the notion of an increase in the quantity, security and predictability of income being the ‘ultimate “multipurpose” policy instrument’ [14].

Alongside this building evidence, though not necessarily because of it, recent UK governments have committed to a ‘prevention agenda’ [15] in improving population health, with more specific goals incorporated into the 2019 NHS England Long Term Plan [16]. There is longstanding evidence on the need for economic interventions to address population health [17–19] and existing approaches are proving inadequate means of reducing the burden on reactive services, with rates of mental health problems [20] and physical impairments [21] continuing to rise.

**Fig 1:**
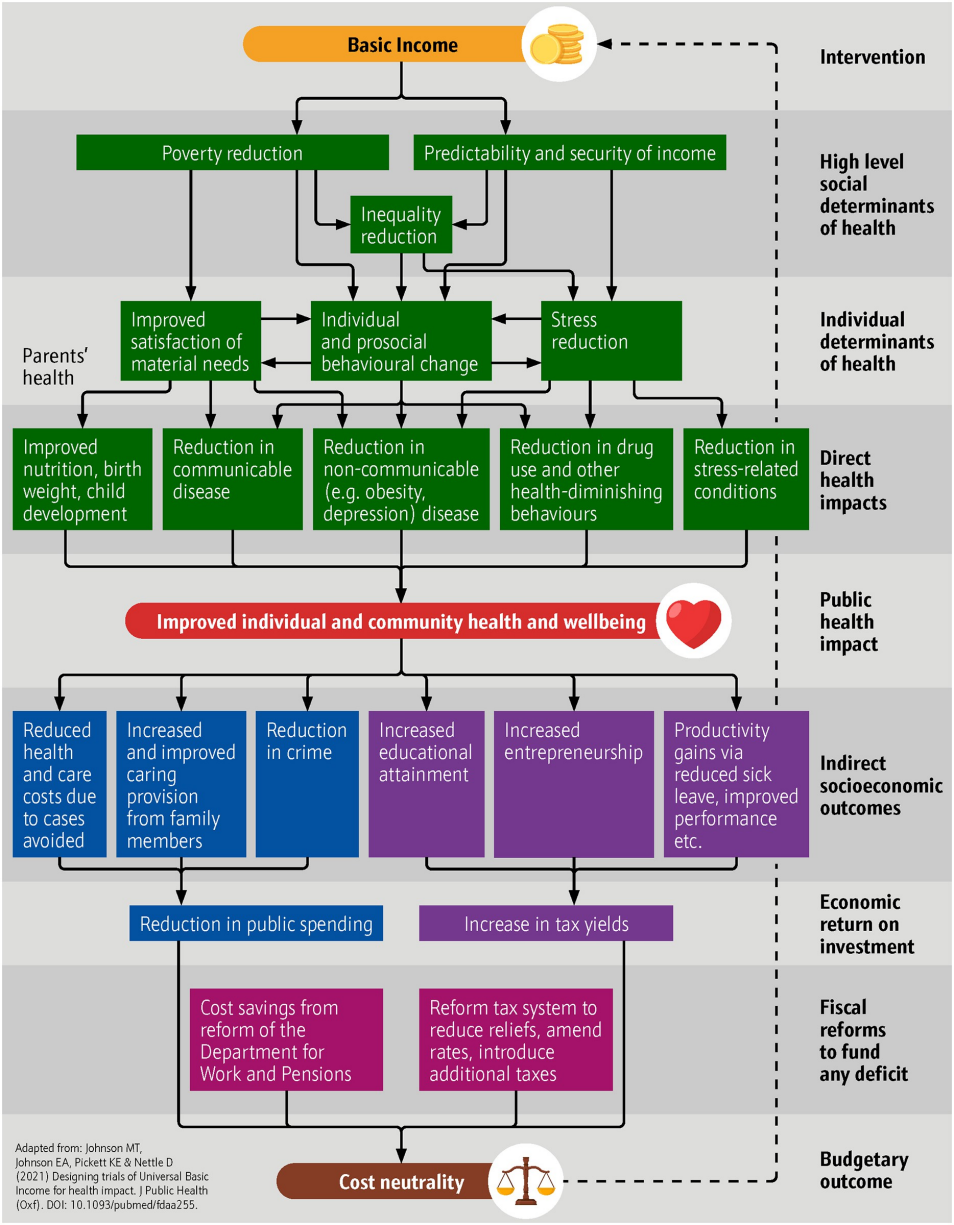
Basic Income model of health impact.

Basic Income (BI) is a largely unconditional, secure, regular payment to all permanent residents to support basic needs regardless of employment status or income. It has been proposed, including by some of the authors of this paper, [22] as an upstream intervention to improve population health by increasing income size and security. We anticipate that Basic Income would work through the following pathways shown below in Fig 1.

In this paper, we combine analysis based on a ‘within-between’ model–which is intended to support causal inference from observational data–using data from both Understanding Society: The UK Household Longitudinal Study (UKHLS) and the Family Resources Survey (FRS) to microsimulate the effects of three proposed Basic Income schemes on mental and physical health among UK 18+ adults. The UKHLS analysis can be found in a working paper titled *Examining the relationship between income and both mental and physical health among adults in the UK*, available at https://doi.org/10.17605/OSF.IO/SKPYB.

We seek to answer the following questions:

1. To what extent would cases of mental and physical health problems be affected by Basic Income schemes?
2. What is the likely social return on investment resulting from the health effects of Basic Income schemes in terms of quality-adjusted life years (QALYs) and their UK-relevant willingness to pay value?
3. What are the likely cost savings for the NHS and personal social services as well as patients?

In answering these questions, we build on previous work both within our team and that of others. Our own previous work [7, 8, 23] has focused on mental health among adolescents and young adults, while that of others has generally excluded physical health [24]. This study therefore fills several important gaps in the literature.

## Methods

### Study design and participants

The modelling of the relationship between health variables (explained in detail below) and net equivalised household income (with other individual and household characteristics used as control variables) is carried out using Waves 1–12 of Understanding Society: The UK Household Longitudinal Study (UKHLS), with data collected between 2009 and 2022 [25]. The UKHLS draws on interview data from approximately 40,000 households per wave to present socioeconomic, demographic and health data of individuals living in private households in the UK. The UKHLS modelling in this paper uses a balanced panel of individuals with data for all 12 waves of the UKHLS, which is a much smaller sample at just under 7,000 individuals per wave. In the appendix of this paper, we also present descriptive statistics for an unbalanced panel consisting of all adult individuals with at least two consecutive waves of UKHLS data. The characteristics of our UKHLS balanced panel sample are shown in Table 1, below.

**Table 1:** UKHLS balanced panel sample characteristics.

	Mean	Observations (individuals n = 6,649)
**Health**		
SF-12 Mental Component Summary (MCS) score	50·47	79,788
SF-12 Physical Component Summary (PCS) score	50·18	79,788
SF-6D score	0·7857	79,788
**Age**		
18–24	1·98%	79,788
25–34	9·51%	79,788
35–44	17·04%	79,788
45–54	22·21%	79,788
55–64	23·68%	79,788
65–74	19·25%	79,788
75+	6·33%	79,788
**Gender**	**Percentage**	
Female	42·34%	79,788
Male	57·66%	79,788
Ethnicity*		
White British/English/Scottish/Welsh/Northern Irish	90·16%	79,788
White (other)*	3·25%	79,788
Mixed**	1·22%	79,788
South Asian (Indian, Pakistani, Bangladeshi)	2·61%	79,788
Chinese and any other Asian background	0·86%	79,788
Caribbean, African and any other black background	1·61%	79,788
Other	0·29%	79,788
**Country of birth**		
Born in the UK	92·30%	79,788
Not born in the UK	7·70%	79,788
**Nation within UK**		
England	87·70%	79,788
Scotland	7·60%	79,788
Wales	3·70%	79,788
Northern Ireland	2·60%	79,788
**Marital status**		
Single, never married	11·60%	79,788
Cohabiting	9·46%	79,788
Married/in a civil partnership	63·96%	79,788
Divorced	8·16%	79,788
Widowed	5·10%	79,788
Other	1·70%	79,788
**Occupational classification (NS-SEC) based on current job**		
Managerial and professional	30·50%	79,788
Intermediate	9·22%	79,788
Small employers and self-employed	5·31%	79,788
Lower supervisory and technical	3·39%	79,788
Semi-routine and routine	11·95%	79,788
Not employed	38·78%	79,788
Missing	0·86%	79,788
Labour market status		
Employed	52·07%	79,788
Unemployed	2·14%	79,788
Family care	3·74%	79,788
Full-time student	0·90%	79,788
Long term sick or disabled	2·12%	79,788
Retired	29·70%	79,788
Other	13·47%	79,788
Education: highest qualification		
University degree	34·46%	79,788
Other higher education (e.g. professional qualifications)	15·34%	79,788
A level	17·69%	79,788
GCSE/ O Level	18·27%	79,788
Other qualifications	8·18%	79,788
No qualifications	6·06%	79,788
Household level variables:		
Household income		
Net household monthly income before housing costs (unequivalised)	£3,993·02	61,948
Net household monthly income before housing costs (equivalised)	£2,411·80	61,948
Housing costs	£266·97	61,948
Household tenure		
Own (outright or with mortgage)	75·19%	61,948
Renting: local authority or housing association	9·94%	61,948
Renting: private landlord	6·41%	61,948
Other	8·46%	61,948
Household composition		
Average number of adults using OECD definition (age 14+)	2·08	61,948
Average number of children using OECD definition (age 0–13)	0·38	61,948

The Landman Economics Tax Transfer Model, running on data from the 2021–22 Family Resources Survey (FRS) dataset, is used to microsimulate net equivalised household incomes under three different Basic Income schemes, described below. The regression coefficients from our analysis of the relationship between health variables and income, controlling for other household and individual characteristics, are used to predict the improvements in population health arising from each Basic Income scheme.

We used this as the basis for our microsimulation’s synthetic population, weighted for additional characteristics found in the 2021–22 FRS dataset. The characteristics of the 2021–22 FRS population used in the Tax Transfer Model are shown in Table 2 below.

**Table 2:** Microsimulation model population characteristics (Family Resources Survey).

	Mean (n = 27,468)
**Age**	
18–24	4·75%
35–44	15·08%
45–54	15·65%
55–64	18·84%
65+	33·23%
**Gender**	**Percentage**
Female	46·80%
Male	53·20%
Ethnicity*	
White (British/English/Scottish/Welsh/Northern Irish/other)	91·06%
Mixed**	0·97%
South Asian (Indian, Pakistani, Bangladeshi)	3·61%
Chinese and any other Asian background	1·07%
Caribbean, African and any other black background	2·01%
Other	1·29%
**Country of birth**	
Born in the UK	86·59%
Not born in the UK	13·41%
**Nation within UK**	
England	73·89%
Scotland	10·86%
Wales	4·88%
Northern Ireland	10·37%
**Marital status**	
Single	17·68%
Cohabiting	11·27%
Married/in a civil partnership	54·50%
Divorced	7·19%
Widowed	7·16%
Other	2·20%
**Occupational classification (NS-SEC) based on current or last job**	
Managerial and professional	42·15%
Intermediate	14·04%
Small employers and self-employed	8·36%
Lower supervisory and technical	5·77%
Semi-routine and routine	23·45%
Never worked and long term unemployed	0·40%
Full-time student	2·02%
Not classified	3·81%
**Labour market status**	
Employed	53·54%
Unemployed	1·42%
Family care	1·91%
Full-time student	1·29%
Long term sick or disabled	5·98%
Retired	32·19%
Other	3·67%
**Education**	
University degree	33·64%
Other higher education (e.g. professional qualifications)	13·08%
A level	16·09%
GCSE/ O Level	17·48%
Other qualifications	5·14%
No qualifications	14·57%
**Number of households**	16,364
**Household income**	
Net household monthly income before housing costs (unequivalised)	£2,818·90
Net household monthly income before housing costs (equivalised)	£1,857·21
**Housing costs**	£265·35
**Household tenure**	
Own (outright or with mortgage)	75·19%
Renting: local authority or housing association	9·94%
Renting: private landlord	6·41%
Other	8·46%
**Household composition**	
Average number of adults using OECD definition (age 14+)	1·78
Average number of children using OECD definition (age 0–13)	0·33

### Procedures

We used the Tax Transfer Model to microsimulate the distributional household income effects of three Basic Income schemes and, with a new module, the health effects resulting from that redistribution. The schemes were broadly designed to provide pathways towards attaining the Minimum Income Standard (MIS). MIS is the income needed by different types of households to reach a socially acceptable living standard, as determined by members of the public with support from experts [26]. The three Basic Income schemes modelled were the following, based on those in Reed et al. [27] and uprated according to the latest Minimum Income Standard:

**Scheme 1—Starter (per week): £50 per child; £75 per adult over 18 and under 65; £205 per adult aged 65+**
  Scheme 1 is fiscally neutral in static terms and does not include savings and returns from investment elsewhere as a result of its introduction. It is affordable within the current fiscal context in the UK. No additional funding from the Exchequer and no net increase in taxation is required.
**Scheme 2—Intermediate (per week): £75 per child; £185 per adult under 65; £205 per adult aged 65+**
  Scheme 2 is a mid-point between the lower and higher levels. It is not fiscally neutral but can be funded by a range of means.
**Scheme 3—MIS level (per week): £100 per child; £295 per adult under 65; £295 per adult aged 65+**
  Scheme 3 ensures that all families reach the MIS level. It has a significant up-front cost but can be funded by a range of means.

These schemes would minimise losses for low-income households and the amount of disruption involved in moving to a new income support system, while enjoying broad public support. For instance, these schemes have been found to have support among critical ‘red wall’ voters in Wales and the Midlands and North of England [28].

The level of payments in each of the schemes is based on existing analysis by Reed et al., [27] uprated according to the UK Consumer Prices Index (CPI). Some of the components of MIS, such as food and energy, have experienced inflation rates much higher than the overall CPI average of around 10%. This means that the MIS scheme, for example, has risen by more than 28% compared with our 2022 report [27].

In the schemes, the main non-means-tested benefits in the UK benefits system (Child Benefit and the State Pension) are replaced by Basic Income. Basic Income payments are counted as unearned income to calculate Universal Credit–the main UK means-tested benefit for people on low incomes–which results in a one-for-one replacement. However, a small disregard is applied for schemes 1 and 2 so that low-income individuals and families are better off under Basic Income than the baseline system. In Scheme 3, payments are sufficiently high that no disregard is necessary. Schemes 2 and 3 are not fiscally balanced (i.e. increases in tax revenue do not match the cost of Basic Income expenditure, net of any reductions in other benefits), but the costs are static and do not take account of any behavioural changes or savings that may be made in other areas as a result of improved productivity, reduced crime etc.

Following this economic microsimulation, we estimated effects on cases of depressive disorders and physical health problems (as measured by self-reported SF-12 Mental Components Summary [MCS-12] and Physical Component Summary [PCS-12] scores), quality-adjusted life years (QALYs) (calculated from SF-12 to SF-6D score) and willingness to pay value gained, as well as direct NHS, personal social services and patients’ associated costs savings with regard to depressive disorders only.

**Fig 2:**
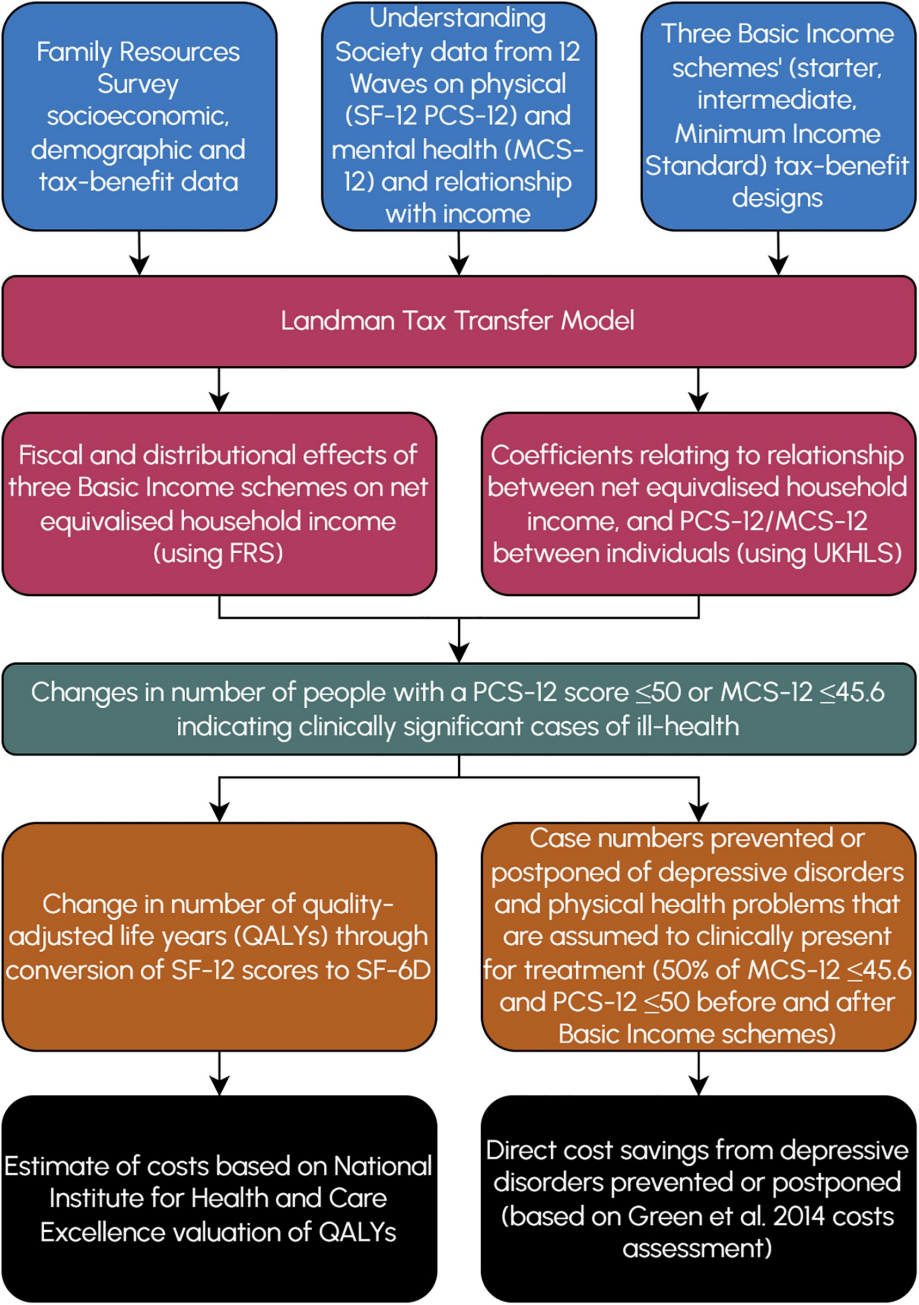
Modelling process diagram.

Fig 2 shows a graphical representation of the modelling process.

### Statistical analysis and outcomes

Based on work in an existing paper, we used a within-between model (estimated using the UKHLS balanced panel shown in Table 1) to examine associations based on both increases or decreases in individuals’ income compared with their average over time (the within component) and individuals’ average income compared with the average of the population (the between component) on the one hand and health on the other. Net equivalised household income is the sum of net monthly incomes from all household members, adjusted by the OECD-modified equivalence scale [29] to account for households of different sizes and composition. In all results, we mean household income, even when we refer to an individual’s income. Following the definition in national statistics, a household is defined as one person living alone, or a group of people (not necessarily related) living at the same address who share cooking facilities and share a living room, sitting room or dining area. A household can consist of a single family, more than one family or no families in the case of a group of unrelated people.

We believe that this model incorporates a number of key income-based drivers of health, including temporary and permanent income shocks, objective inequality and subjective social status inequality, to greater or lesser extents. The significance of income variations on anxiety and depression has been indicated by recent natural experiments, which have shown that reduction in available resources has immediate and significant mental health impacts [30]. It does not, however, capture what we anticipate through our model of impact to be very substantial benefits from Basic Income of increased security of income and protection from destitution for a very large proportion of the population in even relatively highly paid jobs.

Using Stata 15 [31] to run this model, as in our previous studies, [23] we found that increases in income and higher average income is associated with better mental and physical health.

These findings fed through to our microsimulation modelling. Contrasting with our previous project, which used two separate microsimulations (economic and then health) in a hybrid serial arrangement, here we used the coefficients from the “between” components of the UKHLS income model to forecast changes in population health in the FRS, based on the changes in net income arising from the counterfactual net equivalised household income distributions as microsimulated using the Landman Economics Tax Transfer Model (TTM).

The TTM is a microsimulation model of the tax-benefit system of the UK and its constituent countries. The TTM uses data from the Family Resources Survey (FRS) and/or UKHLS [23, 27] to analyse the impact of direct taxes, Universal Credit and other means-tested benefits, and the Living Costs and Food Survey (LCF) to analyse the impact of indirect taxes. The information in FRS or UKHLS and LCF allows payments of direct taxes and receipts of welfare payments to be modelled for each family in the surveys using either the current tax-benefit system and an alternative system to establish the impact for reform. The TTM produces outcomes including the following:

- Aggregate costings of each Basic Income system in terms of amount received in direct and indirect personal taxes, and amount paid out in welfare payments
- Distributional impacts of reforms compared to base system, such as change in incomes in cash terms and as a percentage of weekly income in the base system
- Proportions of exchequer savings/costs due to a particular reform or set of reforms paid for by/going to particular sections of the population
- Average impact of reforms on the household incomes of particular types of individuals, such as children, working age adults and pensioners
- Winners and losers from a particular reform or set of reforms grouped according to size of cash gain or size of percentage gain
- Impact of reforms on overall inequality of disposable incomes through the Gini coefficient
- Impact of reforms on household, adult and child poverty rates using definitions such as proportion of children below 60% of median income
- Changes in Marginal Deduction Rates (MDRs)–net gain to people in employment from an extra pound of earned income, taking into account income tax and National Insurance Contributions paid on extra gross earnings as well as the Universal Credit taper

Crucially, the TTM can now be used for modelling the impacts of changes in income on mental and physical health using empirical relationships established through UKHLS (10.17605/OSF.IO/SKPYB.) data. The distributional results (and many of the other results such as policy costings) from the TTM can be broken down according to a number of population characteristics, including: household type (e.g. single adults, lone parents, couples without children, couples with children); working age adults/pensioners; age group; ethnicity; disability status of adults or children in the household; country/region; housing tenure type; employment status of adults in the household, and decile or quintile of net incomes.

The TTM can be used to analyse changes to most direct and indirect tax policies and welfare policies in the UK and constituent countries, except for policies where the FRS/UKHLS/LCF datasets do not contain enough information on the impact of the policy on individuals and families for the policy to be modellable, such as, for example, the impact of sanctions on UC or other benefit claimants. The TTM also takes account of partial (i.e. less than 100% of the population) take-up of Universal Credit, Pension Credit, Housing Benefit and other benefits [32].

In this study, we used the between-individual components because Basic Income would be a permanent rather than transitory change in income levels. The within coefficients mainly capture transitory variations in income. There is also an essential aspect of between-individual income which has subjective and prospective impacts on individuals. There is substantial and increasing evidence (see, for example, Wilkinson and Pickett’s *Spirit Level* [33] for a summary) that inequality is causally related to outcomes, not just objective income levels. The between measure captures this (at least to some extent).

The TTM runs on Python 2.7. Detailed methodology for the TTM is available in Portes and Reed, [34] and Women’s Budget Group, Runnymede Trust, RECLAIM & Coventry Women’s Voices [35]. For uses of the TTM for microsimulation and varying assumptions, examples and details can be found in Reed and Portes [36] and Harrop and Reed [37].

We examined the effects on case numbers of depressive disorder and physical health problems prevented or postponed, QALYs and associated UK-relevant willingness to pay (WTP) social value gained, Years of Life Gained (YLG), as well as direct NHS, personal social services and patients’ associated costs savings based on depressive disorders alone. Because this study is concerned with the public health impact of Basic Income, we have used NICE valuation of a QALY (£30,000), [38] rather than the current Treasury Green Book valuation, which is £70,000 in 2020/21 prices [39].

The estimated reductions in healthcare costs and increases in QALYs are calculated using the FRS data based on the coefficients from the UKHLS health-income model. When estimating how an increase in net incomes translates into a gain in QALYs for a given person in the UKHLS sample, we assume that the implementation of a tax-benefit change gives rise to a permanent change in incomes and hence a permanent change in SF-6D score for each sample member. We calculate the change in SF-6D for each individual based on their predicted change in SF-12 scores in the income to SF-12 regressions. In other words, we assume that a tax-benefit scheme policy is introduced permanently. The change in SF-6D for each sample member is then translated into a change in QALYs using the mapping between the SF-6D metric and QALYs as defined by SF-6D’s creators [40]. This change in QALYs is summed across the UKHLS sample, grossed up to population level and treated as an annual impact of the policy. These annual impacts can be summed over the number of years that the policy is in operation. It should be noted that we use SF-6D rather than SF-6Dv2 which was not available at the time of undertaking the study.

To estimate the confidence intervals in the UKHLS model, we used a bootstrapping procedure on the 12-wave UKHLS balanced panel sample, randomly drawing (with replacement) from the sample of individuals with a full 12 waves of observations in the data. The bootstrapping procedure used 1,000 repetitions to estimate the upper and lower 95% confidence intervals for the income variables in the MCS-12 and PCS-12 regressions. The estimated changes in PCS-12 and MCS-12 scores arising from modelled changes in income resulting from the introduction of each Basic Income scheme were then converted into estimated changes in SF-6D score using the proprietary conversion algorithm from QualityMetric [41] and University of Sheffield [40].

Costs for anxiety and depression treatment were informed by the usual care arm of the CADET randomised control trial [42]. We estimated and report two different cost perspectives 1) the UK NHS and Personal Social Services (Third Party Payer) perspective, and 2) A broader perspective that included resource use from primary/Community Care (e.g. GP, Mental Health worker, Social worker), Secondary Care (e.g. Hospital admissions, Psychiatric rehab ward, Outpatient appointment, social care (e.g. Daycare centre, drop in a club), informal care from friends/relatives (e.g. Hours per week help from friends/relatives), patient other costs (e.g. OTC medications, Travel costs) to estimate the total cost of anxiety and depression. We uprated all costs to 2023 British pounds using the CPI. We further assumed that only half of the modelled individuals that reported symptoms of anxiety or depression would seek treatment and thus incur healthcare costs. This assumption was roughly informed by the Adult Psychiatric Morbidity Survey [20]. We extended this assumption to cases of physical health problems on the basis that producing conservative estimates is likely to have fewer real-world negative effects when assessing the cost-benefit of a policy like Basic Income than the alternative.

We controlled for whether individuals were depressed or had symptoms indicating a physical health problem in the previous period as well as sex, age, ethnicity, whether the individual was born in the UK, region, rurality, highest qualification, marital status, employment status and attrition.

Health variables of interest were the following:

#### SF-12

The SF-12 (v2) survey [43] is a widely used tool to assess an individual’s health-related quality of life, generating two summary scores: the Physical Component Summary (PCS-12) and the Mental Component Summary (MCS-12). We use an overall SF-12 score as well as MCS and PCS scores as outcome variables using our within-between model. Additionally, we create a dichotomous variable for cases of depressive disorder which takes the value of 1 if the individual’s score is ≤45.6 and 0 if it is ≥45.7 [44]. In the case of the PCS-12, we use a threshold for clinically significant symptoms of a physical health problem of ≤50 [45]. The items comprising SF-12 can be found in the UKHLS Wave 12 questionnaire [46]. The scoring system used can be found in Ware et al. [43].

#### SF-6D (SF-12)

SF-6D [47] is a preference-based measure of health which enables the calculation of QALYs and therefore economic evaluation and cost-effectiveness of interventions. Originally developed to derive utility values from items in the SF-36 measure of health, an index based on seven items in six dimensions from SF-12 was created in 2004, [48] denoted by SF-6D (SF-12). The dimensions cover, with UKHLS variables in parentheses, physical functioning (scsf2a), role limitations (scsf3a and scsf3b), social functioning (scsf7), pain (scsf2a), mental health (scsf6c), and vitality (scsf6b). Each dimension has between three and five levels with 7,500 possible health states based on one selection in each dimension [48]. The valuation study was undertaken in the UK.

We used software from QualityMetric [41] to convert SF-12 scores into SF-6D scores which were then used to calculate the change in QALYs for the UK population arising from redistribution of income through the Basic Income schemes with their accompanying funding mechanisms.

#### Quality-Adjusted Life Years (QALYs)

QALYs are a widely recognized standardised measure of health outcomes, including those that are not directly comparable, commonly used in health economics and health technology assessment [49, 50]. QALYs capture not only the increased life expectancy resulting from an intervention but also the improvements in health-related quality of life, with a year in perfect health assigned a QALY value of 1, while a year of less-than-perfect health receives a value less than 1. Patient wellbeing is assessed in the physical, social, and psychological domains, and QALY weights are empirically assigned to each dimension. In the context of this study, QALYs enable policymakers and stakeholders to compare the cost-effectiveness of Basic Income with other health interventions and make informed decisions about resource allocation to maximise health gains. Additionally, QALYs incorporate a time dimension, useful to consider the duration of health improvements resulting from long-term policies.

Each QALY has been assigned a value of £30,000 based on NICE guidance which suggests that between £20,000 to £30,000 per QALY gained as a result of an intervention may be deemed cost effective [51]. The Treasury’s Green Book proposes a much higher value of £70,000 but generally relates to other non-health expenditure interventions such as those related to transport [39]. In order to consider the broader impact of the intervention for assessment of Basic Income as a welfare policy intervention, the results we produce can be multiplied by 2.33.

#### Years of Life Gained (YLG)

In order to calculate differences in life expectancy, we use multipliers (conditional on gender and age) for men and women of each year-of-age group in the FRS data. The multipliers are derived from McNamara et al. [52]. The gender- and age-specific multipliers are used to produce an estimate for years of life expectancy gained, conditional on QALYs gained, for men and women in each age group. These are then summed across the whole (grossed-up) FRS population to produce estimates for total Years of Life Gained (YLG) for each Basic Income scenario.

### Role of the funding source

The funder of the study had no role in study design, data collection, data analysis, data interpretation, or writing of the report.

## Results

Table 3 shows the number of cases of anxiety and depression and of clinically significant physical health symptoms prevented or postponed under each scheme. Between 124,000 (95% CI: 86,000 to 150,000) and 1.005 million (95% CI: 845,000 to 1.402 million) cases of depressive disorders and 118,000 (95% CI: 70,000 to 156,000) to 1.042 million (95% CI: 881,000 to 1.612 million) cases of physical health problems could be prevented or postponed each year depending on the scheme.

**Table 3:** Modelling results indicating cases of depressive disorders and physical health problems with confidence intervals in parentheses among 18+ adults prevented or postponed in 2023.

	Cases of depressive disorders prevented or postponed	Cases of physical health problems prevented or postponed
**Scheme 1**	124,000 (86,000–150,000)	118,000 (70,000–156,000)
**Scheme 2**	537,000 (446,000–747,000)	548,000 (457,000–833,000)
**Scheme 3**	1,005,000 (854,000–1,402,000)	1,042,000 (881,000–1,612,000)

Table 4 shows that between 129,000 (95% CI: 86,000 to 172,000) and 655,000 (95% CI: 440,000 to 870,000) QALYs could be gained, valued at £3.87 billion (95% CI: £2.58 billion to £5.16 billion) to £19.65 billion (95% CI: £13.21 billion to £26.10 billion). The savings are independent of the cost of the investment, the framework for which has been outlined previously [27].

**Table 4:** Modelling results indicating the estimated number and value of QALYs gained with confidence intervals in parentheses as a result of each Basic Income scheme in 2023.

	Number of QALYs gained	Value of QALYs gained (£30,000 each)
**Scheme 1**	129,000 (86,000–172,000)	£3·87 billion (£2·58 billion–£5·16 billion)
**Scheme 2**	375,000 (252,000–499,000)	£11·28 billion (£7·57 billion–£14·98 billion)
**Scheme 3**	655,000 (440,000–870,000)	£19·65 billion (£13·21 billion–£26·10 billion)

Table 5 shows that between 169,000 (95% CI: 113,000 to 226,000) and 860,000 (95% CI: 578,000 to 1.143 million) years of life could be gained in 2023.

**Table 5:** Modelling results indicating the estimated Years of Life Gained (YLG) with confidence intervals in parentheses as a result of each Basic Income scheme in 2023.

	Number of Years of Life Gained (YLG)
**Scheme 1**	169,000 (113,000–226,000)
**Scheme 2**	494,000 (331,000–656,000)
**Scheme 3**	860,000 (578,000–1,143,000)

Table 6 shows NHS and personal social services costs savings as well as total costs savings [42] associated with the cases of depressive disorders prevented or postponed in 2023, which are estimated at between £563 million (95% CI: £392 million to £686 million) and £4.579 billion (95% CI: £3.893 billion to £6.391 billion) assuming 50% of depressive disorders cases would be diagnosed and treated in the baseline.

**Table 6:** Modelling results indicating per year depressive disorders cost savings with confidence intervals in parentheses from different perspectives.

	NHS and personal social services cost savings assuming 50% of cases diagnosed and treated	Total (including patients’ related) cost savings assuming 50% of cases diagnosed and treated
**Scheme 1**	£126 million (£88 million–£154 million)	£563 million (£392 million–£686 million)
**Scheme 2**	£549 million (£455 million–£763 million)	£2·449 billion (£2·032 billion–£3·404 billion)
**Scheme 3**	£1·026 billion (£872 million–£1·432 billion)	£4·579 billion (£3·893 billion–£6·391 billion)

## Discussion

These microsimulation findings indicate that Basic Income schemes could provide a significant benefit to the mental and physical health of adults, a large social return on investment and substantial savings for the health and social care system as well as in costs incurred by patients. The three Basic Income schemes reduce income inequality and increase the average incomes of the lowest quintile (poorest 20%) of household net equivalised incomes in particular. It is this boost to income for low-income households which drives the projected increases in population health arising from the introduction of Basic Income.

Previous studies in a UK context have estimated impacts focusing on mental health alone and/or particular age groups. Compared with Parra-Mujica et al. [7] and Chen et al., [23] which also use UKHLS data to examine the relationship between income and mental health and microsimulate impacts based on Basic Income schemes respectively, this paper expands analysis to all adults, rather than focusing on 16/18- to 24-year-olds, and covers both mental and physical health. Physical health, in particular, has not been assessed or modelled comprehensively in previous studies. Similarly, Thomson et al., [24] which was published after our findings had been produced and drafted, focuses exclusively on mental health and adults aged 25–64. Thomson et al.’s analysis also uses an alternative measure of mental health, GHQ-12, whereas SF-12 has an established and rigorous process of translation to QALYs using NICE guidelines. Green Book value can be produced by using a 2.33 multiplier.

This study uses validated measures from a large-scale, long-running and nationally significant household longitudinal study. It also builds on an economic microsimulation model that has previously been subject to peer-review. For the first time, it has provided an estimate of potential health impacts of Basic Income schemes across the adult population. It is, however, based on observational data and assumes that low income is causally related to depressive disorders and physical health problems and that increasing income can fully reverse the risk. The association between income and mental health has been shown in experimental and observational studies. Recent evidence from the cost-of-living crisis supports the assumption that reduction in non-committed money has a causal effect on mental health [30]. However, the heterogeneity of cash transfer schemes and other policies intended to redistribute income and the heterogeneity of reported mental health outcomes make evidence synthesis difficult. Large, representative trials of Basic Income that capture comprehensive and comparable data in the real world are crucial [53]. There remains an opportunity to model the health impacts of changes through all pathways identified in Fig 1 on all major disease types. This would enable much greater specificity in the types of health problems addressed and associated savings, including when extrapolating from trials.

As discussed above, we believe that the ‘between’ coefficient from our analysis most closely resembles the impact of a permanent change in income from a Basic Income, also reflecting the cumulative and subjective impacts of inequality. There remains, however, a challenge with regard to income quintiles being the average across the country, as differences in living costs might have a significant impact on people’s day-to-day experience and material conditions. We are currently examining a measure that would more accurately reflect the effect of people’s uncommitted income while also being usable within microsimulation.

It is important to note that the cost-of-living crisis and the high inflation period in the UK and much of the wider world in 2023 has compressed, and is likely to compress further, household incomes, accelerating the situation in public health further by negatively affecting the pathways set out in Fig 1. This suggests that our modelled estimates are conservative and that research on Basic Income policies is continuing to build both in relevance and both political and public health salience. There is evidence to suggest that the redistributive impacts of basic income schemes will benefit women more than men, who have lower incomes on average, and lower income groups more than higher income groups [27]. It is also assumed that additional needs relating to disability will be provided by supplementary conditional payments [54]. There is no evidence that basic income produces negative labour market participation outcomes. Indeed, basic income reduces the participation tax rates associated with negative income tax schemes–that is, the loss of benefits as income from work increases–identified by Martinelli as particularly affecting lower income households [55].

The findings have relevance to policymakers in indicating that upstream economic interventions have the potential to improve population health, reduce the burden on downstream, reactive health services, and address the long-term increase in both the prevalence of long-term health conditions and impairments and the associated health and social care costs. Clinicians are increasingly faced with patients presenting with problems that are fundamentally socioeconomic in nature, or the result of socioeconomic factors. Given that they are asked by government to take part in welfare claim and other social processes, there is a need for clinicians to make their voices heard in relation to policy development to ensure that they can continue to do their jobs effectively and support better health and wellbeing for their patients.

Again, with caveats regarding the likely underestimate of impact, the savings from NHS and patients’ related costs could pay the full economic cost for between 7,481 (under Scheme 1) and 61,184 (under Scheme 3) additional hospital-based band-5 nurses, including salary and all other associated costs and overheads, per year [56]. Physical health NHS and personal social services savings are more difficult to calculate. However, it is reasonable to anticipate that they may be even higher, given that the 2022/23 clinical commissioning groups (CCG) budget for mental health, learning disability and dementia services in England is just £13.3bn, or 13.8% of the total [57].

The methods we have employed take into account any prospective negative impacts from an increase or decrease in income at different points in the income distribution. It is important to note that there is some evidence that increases in income at the top end of the distribution have increasingly diminishing returns, such that there may actually be a decrease in health at the absolute top end. There is evidence of increased mortality and accidental deaths in US studies of infrequent cash transfers, for example following the annual Alaska Permanent Fund distribution or the biannual Eastern Cherokee payment, perhaps due to increased, stored-up, activity [22]. This speaks to the importance of other pathways within our model of impact, beyond objective income increases, particularly regularity and security of income. There is no reason to believe that such issues would arise in a Basic Income system with distributions occurring weekly or monthly.

We believe that this study represents a first step in establishing much broader health impacts of prospective Basic Income schemes. However, further work is required to understand the impact of such schemes through all of the pathways identified in our model of impact. Experimental studies, while difficult due to the need to replicate Basic Income at large-scale to imitate a full policy effectively, would provide greater certainty with regard to health impacts and policymaker confidence. However, our study indicates that the health savings and returns on investment could be substantial.
